# Early Outcomes of Proximal Humerus Fractures Treated With Proximal Humerus Locking Plate Fixation Using the Deltoid-Split Approach

**DOI:** 10.7759/cureus.110499

**Published:** 2026-06-08

**Authors:** Ajatshatru, Rajesh Arora, Amit Srivastava, Ish K Dhammi, Rakesh Sehrawat, Rahul Kumar, Naveen K Shah, Harsh S Hada

**Affiliations:** 1 Department of Orthopaedics, University College of Medical Sciences and Guru Teg Bahadur Hospital, New Delhi, IND

**Keywords:** deltoid split approach, delto-pectoral approach, functional outcome in proximal humerus fractures, proximal humerus fractures, proximal humerus locking plate

## Abstract

Introduction

The deltopectoral approach is the workhorse approach used for plating of proximal humerus fractures. However, the less invasive and less commonly used deltoid-split approach has shown comparable outcomes in selected cases. Literature on its functional outcomes and complications remains limited, especially in the Indian subcontinent. This study evaluates the results of patients with proximal humeral fractures managed by open reduction and internal fixation (ORIF) with plating using proximal humerus locking plates (PHLPs) performed through the deltoid-split approach.

Materials and methods

This prospective case series was conducted in a tertiary hospital after ethical clearance. Twenty-one patients meeting the inclusion and exclusion criteria underwent ORIF using the deltoid-split approach. Minimum follow-up was at least one year. Functional outcomes were assessed using QuickDASH (Disabilities of Arm, Shoulder, and Hand) and VAS (Visual Analog Scale) scores. Complications such as surgical site infection, wound complications, nerve injury, implant impingement, or failure were recorded.

Results

At 3, 6, and 12 months, mean QuickDASH scores were 24.41, 12.37, and 3.90, and VAS scores were 5.0, 2.94, and 0.2, respectively. All fractures united by nine months. No surgical site infection, wound dehiscence, varus collapse, or implant failure occurred. One case each of implant impingement and malreduction, and two cases of axillary nerve palsy were noted.

Conclusion

Surgical fixation of proximal humerus fractures using the deltoid-split approach provides good recovery with minimal complications. The outcomes are comparable with those achieved using the deltopectoral approach in the literature. It offers tuberosity access with less soft tissue dissection. Surgeons should be accustomed to both approaches and choose the approach according to the fracture configuration.

## Introduction

The proximal humerus fracture is defined as a fracture at or proximal to the surgical neck of the humerus [1,2]. With increasing urbanization and incidence of road traffic accidents, the incidence of proximal humerus fractures is on the rise and is expected to rise several-fold in the coming years. These fractures have a bimodal age distribution, with more than 80% of cases in individuals aged over 50 years. It usually affects elderly females and young males [3]. Many of these fractures are undisplaced two-part fractures, which have a good functional outcome when managed conservatively. The management options for displaced fractures still remain controversial. Surgical fixation can be done after open or closed reduction. Open reduction can be followed by internal fixation using plating, intramedullary nailing, or tension band wiring, whereas fixation after closed reduction can be done by percutaneous K-wires or closed intramedullary nailing or plate fixation (minimally invasive percutaneous plate osteosynthesis) [4]. Open reduction and internal fixation (ORIF) with plating using proximal humerus locking plates (PHLPs) is the usual modality and provides good functional results [5].

The exposure of the fracture site is a challenging task for any surgeon, especially in cases of multi-fragmentary fractures, where distracting muscular forces displace the greater tuberosity proximally and posteriorly and the lesser tuberosity medially [6]. The conventionally used deltopectoral approach requires extensive soft tissue dissection to reach all fragments, especially the greater tuberosity, and is associated with more blood loss and soft tissue injury [7]. The plate is usually placed on the lateral surface of the humerus. The lateral-based “deltoid-split approach,” requiring less soft tissue dissection and providing direct access to the lateral aspect of the humeral head, is nowadays being used by many surgeons [8,9]. However, it has the potential for injury to the vessels of the humeral head and the axillary nerve.

The current study is an endeavor to assess functional outcomes, union rates, and associated complications in patients with proximal humerus fractures operated on using the deltoid-split approach and fixation using a PHLP.

## Materials and methods

This prospective study was conducted in the Department of Orthopedics at a tertiary care hospital after obtaining institutional ethical clearance and formal informed consent from the patients between June 1, 2024, and January 31, 2026. Twenty-one patients with displaced proximal humerus fractures underwent PHLP fixation (PHEELOS; Auxein Medical Pvt. Ltd., Kundli, Haryana, India) using the deltoid-split approach. All patients were operated on by a single consultant-level orthopedic surgeon. Two patients were lost to follow-up.

Nineteen patients who completed a minimum of 12 months of follow-up were evaluated. Only patients who were operated on within three weeks of trauma and had closed fractures were included. Patients with pathological fractures, fracture dislocations, polytrauma, psychiatric illness, systemic illnesses such as diabetes mellitus, malignancy, end-stage renal disease, or inflammatory arthritis were excluded from the study.

All fractures were classified according to Neer’s classification using plain radiographs and non-contrast computed tomography (CT) with 0.6 mm slice thickness [10].

Surgery was performed under general anesthesia, with the patient in the beach-chair position. A longitudinal incision was made starting from the anterolateral tip of the acromion and extending a maximum of 5 cm distally. Non-absorbable sutures (Biobraid; Auxein Medical Pvt. Ltd.) were passed through the insertion sites of the supraspinatus, infraspinatus, and subscapularis tendons to facilitate the manipulation and reduction of the greater and lesser tuberosities. K-wires were used as joysticks for reducing fragments and holding them temporarily. The axillary nerve was palpated; however, its exploration was avoided. The nerve was secured by forming a submuscular tunnel underneath it using a Cobb’s elevator. The plate was passed from proximal to distal, and its position was confirmed under fluoroscopy. K-wires were used temporarily to fix the plate to the humerus. Proximal K-wires also provided an indication of the position of the proximal-most screws. First, a 3.5-mm cortical screw was inserted through the plate, which acted as a positional screw to indirectly reduce fragments to the shaft. Proximally, fixation was achieved using multiple 3.5-mm locking screws. Inferomedial calcar screws were also inserted if metaphyseal comminution was present. The plate was fixed distally using 3.5-mm locking screws. The size of the screws and the reduction of the fragments were confirmed under fluoroscopy. The deforming forces at the tuberosities were counterbalanced using non-absorbable sutures, which had been passed through the insertions of the supraspinatus, infraspinatus, and subscapularis tendons at the beginning of the incision and tied to the plate.

Patients were encouraged to start pendulum exercises on the day after surgery. Active-assisted gentle range-of-motion exercises of the shoulder, including assisted front and side lifts, were also initiated. The patients were encouraged to increase the duration of these exercises on heals of pain. At six weeks after surgery, independent range-of-motion exercises against gravity resistance were encouraged. At three months, passive stretching and resistive exercises were encouraged. However, the rehabilitation protocol had to be individualized in some patients according to their pain tolerance. Sutures were removed on the 14th day, and rehabilitation was continued. The patients were followed up at 6 weeks, 3 months, 6 months, and 12 months, and clinico-radiological examinations were performed at every follow-up visit.

The assessment of the degree of pain was done using the VAS (Visual Analog Scale) score [11], and the QuickDASH (Disabilities of Arm, Shoulder, and Hand) [12] score was recorded at 3, 6, and 12 months to assess functional outcome. Both these tools are open access. Other parameters evaluated during these follow-ups were signs of infection, fracture union (assessed on follow-up radiographs in two planes), implant failure (plate breakage, screw loosening, or screw cut-out), impingement (painful restriction of shoulder motion due to implant prominence), signs of avascular necrosis (AVN), and varus collapse (loss of reduction with a decrease in the neck-shaft angle on follow-up X-rays). Axillary nerve palsy was also assessed.

The IBM SPSS Statistics for Windows, Version 20 (Released 2011; IBM Corp., Armonk, NY, USA), was used to analyze the relationship between subjective measures (QuickDASH and VAS scores) and objective measures, including complications. The complications of the procedure were reported as percentages, and the quantitative data are expressed as mean ± SD. Changes in QuickDASH and VAS scores across the three follow-up periods (3, 6, and 12 months) were analyzed using the Friedman test. A p-value of <0.05 was considered statistically significant.

## Results

Twenty-one patients were considered for the study. However, two patients were lost to follow-up. Hence, 19 patients were included in the study.

Twelve male and seven female patients, with an average age of 43.3 years (range: 18-60 years), were included. Ten patients had sustained fractures due to road traffic accidents, eight due to low-velocity falls, and one due to a high-velocity fall. Two participants (10.5%) had two-part fractures, 11 (57.9%) had three-part fractures, and six participants (31.6%) had four-part fractures. Six patients had fractures on the left side, and twelve on the right side. All patients were right-hand dominant (Table 1).

**Table 1:** Demographic characteristics of the study population (n = 19) Table describing the mean age of the study population, age distribution, gender distribution, distribution according to fractured side, mechanism of injury, and Neer’s classification.

Basic Details	Mean ± SD/Range/n (%)
Mean age in years (range)	43.3 (19 - 60)
Age distribution	
18-30 Years	2 (10.5%)
31-40 Years	4 (21.05%)
41-50 Years	8 (42.1%)
51-60 Years	5 (26.3%)
Gender	
Male	12 (63.2%)
Female	7 (36.8%)
Side	
Right	13 (68.42%)
Left	6 (31.57%)
Mechanism of injury	
RTA High Velocity Trauma	10 (52.63%)
Low Velocity Fall	8 (42.1%)
High Velocity Fall	1 (5.2%)
Fracture type (Neer classification)	
2 Parts	2 (10.5%)
3 Parts	11 (57.9%)
4 Parts	6 (31.6%)

The average time duration between trauma and surgery was six days (range 2-20 days).

At three months’ follow-up, union was achieved in six cases (31.6%), and after nine months, in all 19 cases (100%). None of the patients had varus collapse, postoperative surgical site infection, or screw perforation. There was no evidence of AVN of the humeral head. Axillary nerve palsy was recorded in two cases (10.5%). In one case (5.2%), subacromial impingement, and in one case (5.2%), malreduction were observed. The overall complication rate was 21.05% (Table 2).

**Table 2:** Incidence of complications

Complications	Yes	No
Malreduction	1 (5.26%)	18 (94.73%)
Impingement	1 (5.26%)	18 (94.73%)
Axillary Nerve Palsy	2 (10.52%)	17 (89.5%)
Varus Collapse	0	19 (100%)
Surgical Site Infection	0	19 (100%)

**Figure 1:**
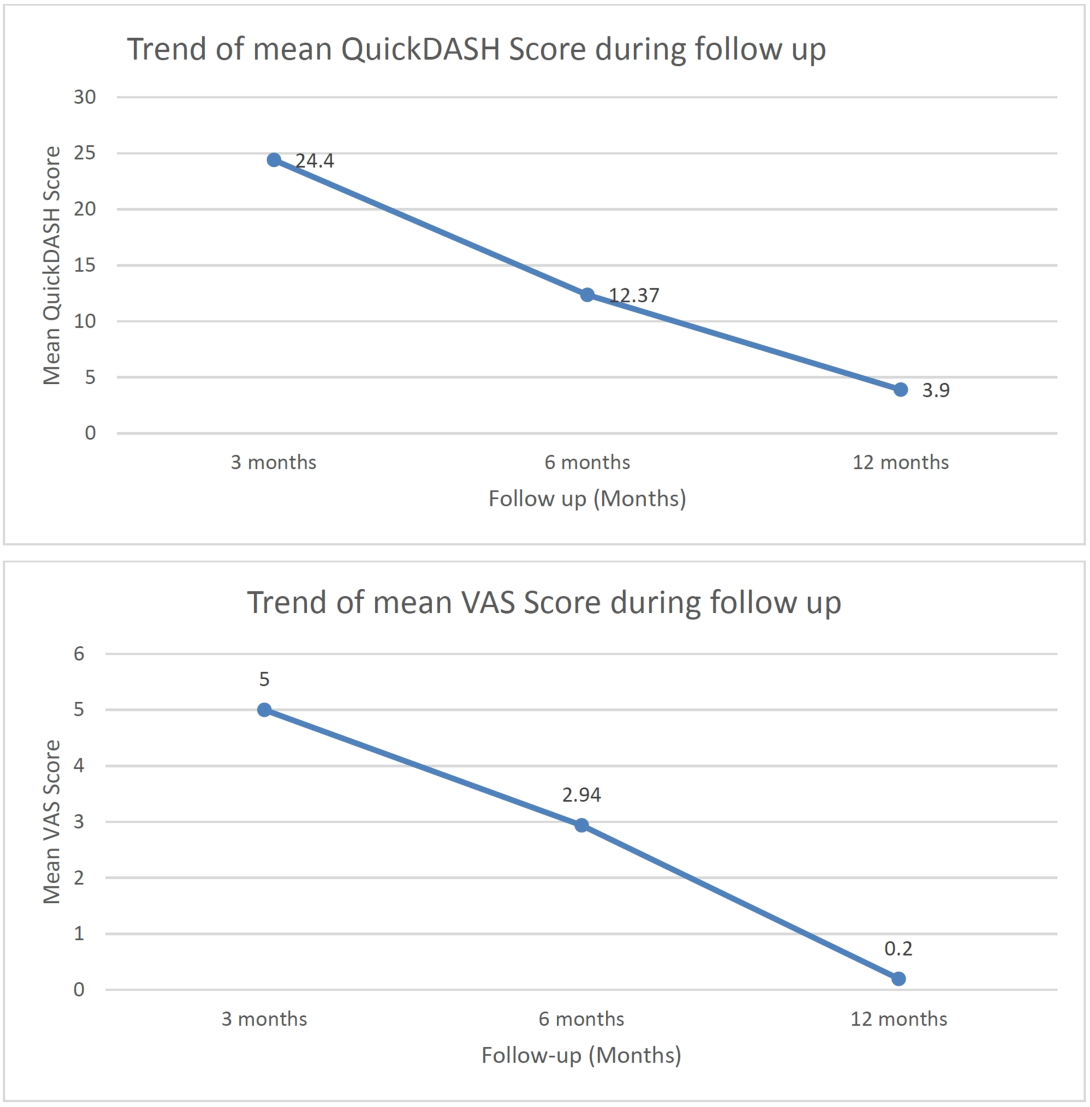
Trend of mean QuickDASH and VAS scores during the follow-up period

**Figure 2:**
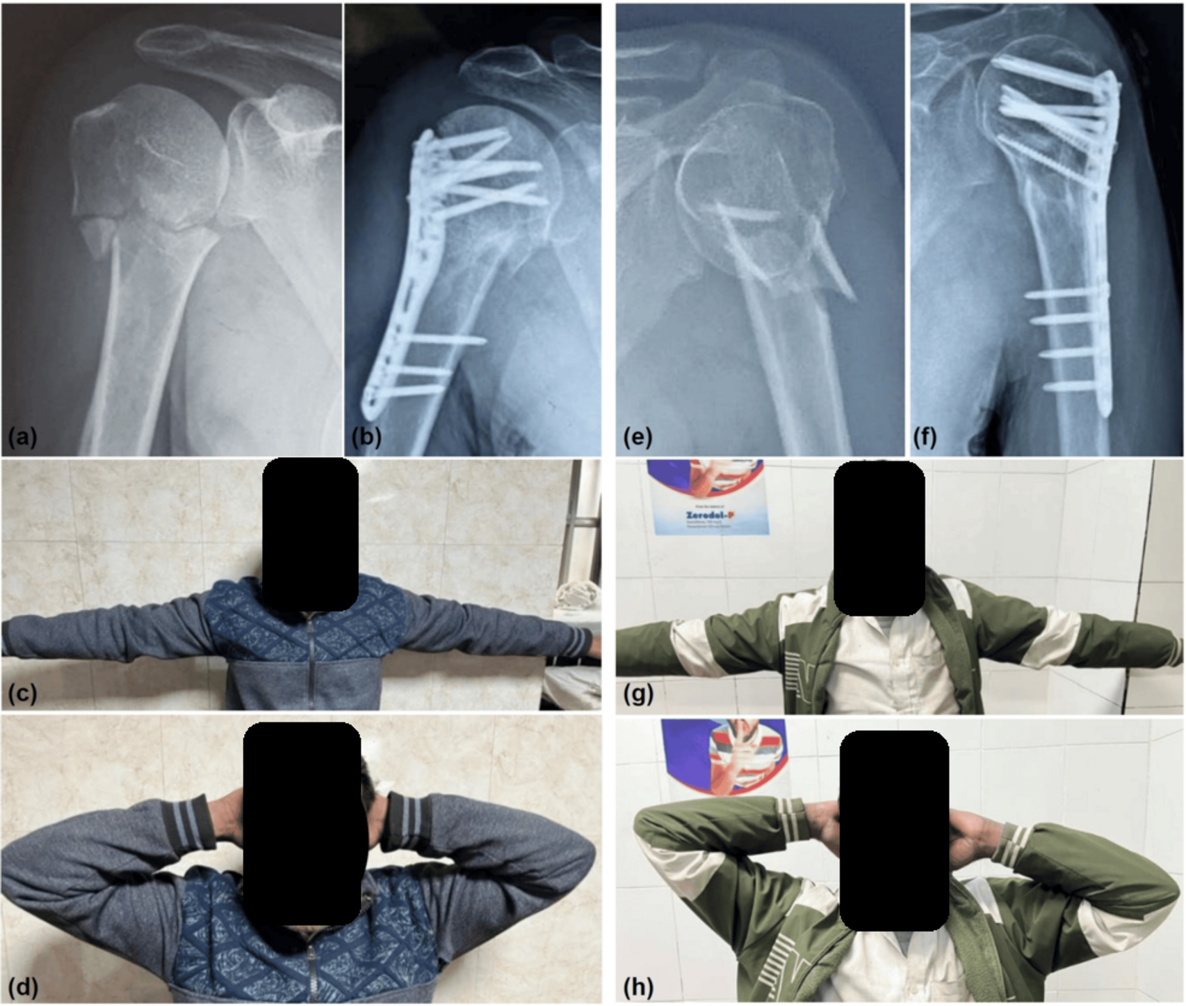
Preoperative radiographs and clinical photographs at the one-year follow-up visit a-b) Preoperative X-ray and X-ray at 1-year follow-up, respectively, of patient no. 15 of the study. c-d) Clinical photographs taken at the 12-month follow-up visit demonstrating the functional outcome of patient no. 15. e-f) Preoperative X-ray and X-ray at 1-year follow-up, respectively, of patient no. 13 of the study. g-h) Clinical photographs taken at the 12-month follow-up visit demonstrating the functional outcome of patient no. 13.

**Figure 3:**
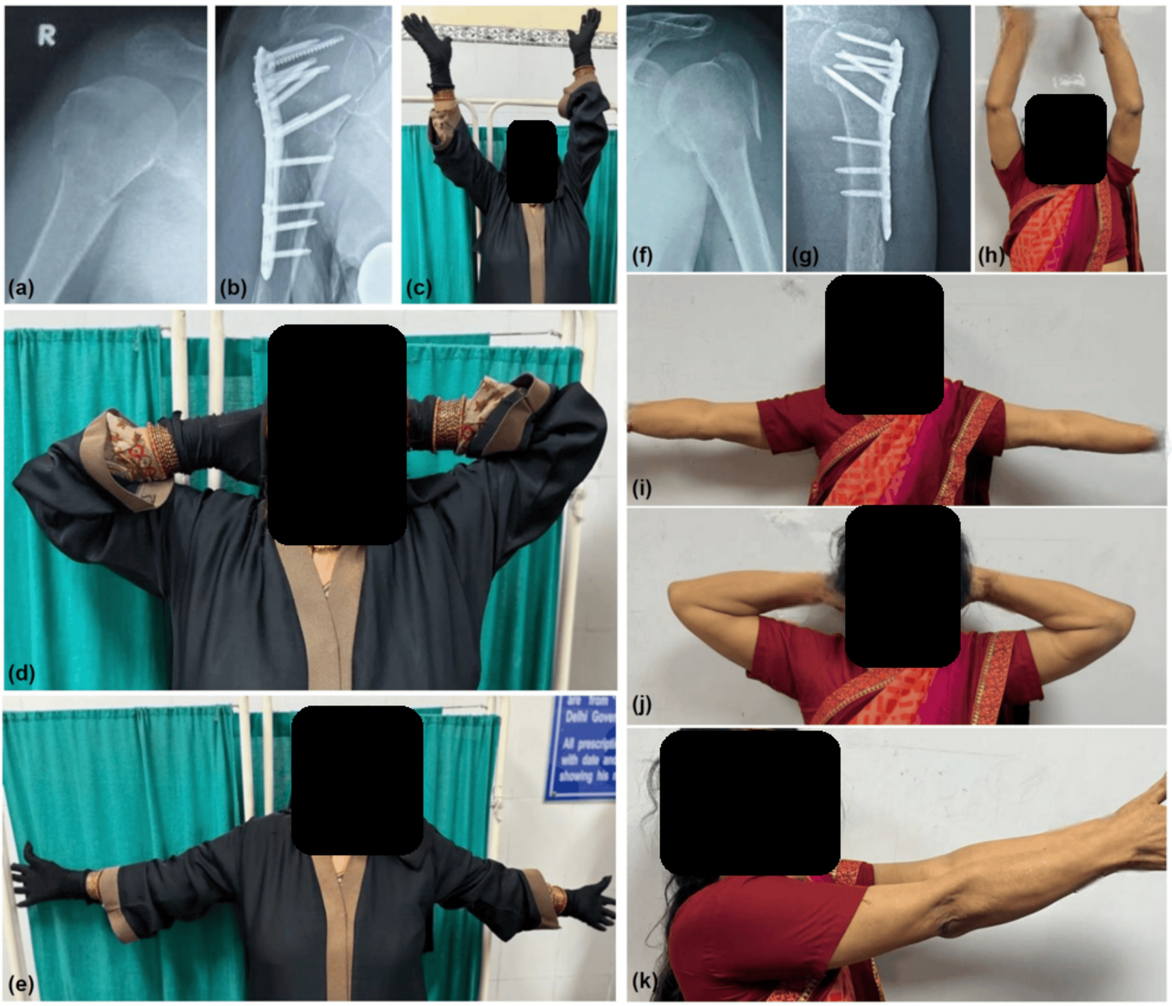
Preoperative X-ray, X-ray, and clinical photographs at one-year follow-up visit a-b) Preoperative X-ray and X-ray at 1-year follow-up, respectively, of patient no. 10 of the study. c-e) Clinical photographs taken at the 12-month follow-up visit demonstrating the functional outcome of patient no. 10. f-g) Preoperative X-ray and X-ray at 1-year follow-up, respectively, of patient no. 3 of the study. h-k) Clinical photographs taken at the 12-month follow-up visit demonstrating the functional outcome of patient no. 3.

The mean QuickDASH score improved progressively from 24.4 (range: 6.8-59.1) at three months to 12.37 (range: 0-45.5) at six months and 3.90 (range: 0-27.3) at 12 months. Across all follow-up periods, functional outcomes were best in two-part fractures, intermediate in three-part fractures, and poorest in four-part fractures (Table 3 and Figures 1-3).

**Table 3:** Mean QuickDASH and VAS scores during follow-up period

Follow-up duration	Mean QuickDASH score	Mean VAS score
3 months	24.4	5.0
6 months	12.37	2.94
12 months	3.90	0.2

VAS pain scores showed a progressive decline over time. At three months, the mean score was 5.0, ranging between 0 and 7.0. The mean score reduced to 2.94 (range 0-4.0) at six months and further to 0.2 at 12 months (range 0-2), indicating near-complete pain relief at final follow-up (Table 3 and Figures 1-3). The Friedman test demonstrated a statistically significant improvement in functional outcome and pain scores over the follow-up period (p < 0.001 for both QuickDASH and VAS scores).

## Discussion

The management of displaced proximal humerus fractures is controversial due to a wide range of options available [13]. There is a broad consensus that undisplaced fractures are managed conservatively and displaced fractures are treated surgically [2,13]. There are many surgical options for management, each having its own set of advantages and disadvantages [13]. PHLP offers the advantage of providing angular stability and facilitating fixation in osteoporotic bone [4]. However, it is debatable whether to use the deltopectoral or deltoid-split approach [8].

We used the deltoid-split approach for plating consecutive proximal humerus fractures presenting to the department that satisfied the inclusion and exclusion criteria. All patients were operated on using the same implant and by the same surgeon. Patients were kept under a guided rehabilitation program and were followed up serially. In 100% of cases, union was achieved by nine months. There was no case of varus collapse, surgical site infection, screw perforation, or AVN.

QuickDASH score and the VAS for pain at 3, 6, and 12 months were used to assess functional outcome. Our results show a progressive improvement in functional scores between 3, 6, and 12 months. At three months, most patients reported moderate disability and residual pain, as reflected by higher QuickDASH and VAS scores. However, at 6 and 12 months, a significant decrease in disability and pain was recorded, highlighting the importance of stable fixation in facilitating rehabilitation and early recovery of function (Figures 2-3). Thus, this declining trend has meaningful clinical implications. By six months, most of the patients reported substantial functional recovery and returned to activities of daily living. Improvement was more marked after 12 months. The reduction in VAS scores demonstrates that, along with recovery of mobility, there was also relief from persistent pain, which is a major factor in patient satisfaction and quality of life.

Significant functional improvement was observed in terms of improved QuickDASH scores, which is consistent with the literature. In a prospective case series of n = 53 patients, Owsley and Gorczyca (2008) concluded that the mean QuickDASH score after ORIF with plating via the deltopectoral approach for proximal humerus fractures was 11 at six months after surgery [14]. In our study, the mean QuickDASH score after ORIF with plating via the deltoid-split approach was 12.37 at six months, suggesting that functional outcomes were comparable when ORIF is performed using either the deltopectoral or deltoid-split approach.

A similar observation was also reported by Aggarwal et al. (2011), who observed significant improvement in shoulder function within the first six months following plating via the deltopectoral approach in proximal humerus fractures [15].

Axillary nerve palsy was noted in two patients. Both of them had a four-part fracture. There was a sensory deficit in the axillary nerve region, and atrophy of the anterior fibers of the deltoid muscle was observed. Despite the presence of palsy, the patients did not experience significant functional compromise. All were able to perform activities of daily living independently and successfully returned to their pre-injury occupation. Implant impingement was noted in one patient. Implant removal was offered to the patient after union; however, she refused the surgical procedure.

Malreduction was observed in one case; infection or non-union was not recorded. Since the follow-up period of this study was only 12 months, it is too early to conclude that there were no cases of AVN. With regard to fracture healing, 17/19 fractures united by six months, and all 19 fractures united by nine months after surgery. This is in agreement with reports by Hertel et al. (2004) and Südkamp et al. (2009), who found that locking plates provide reliable fixation and promote fracture healing in most cases [16,17]. No patient developed infection, which reflects the relatively small incision and limited dissection required in the deltoid-split approach as compared to the deltopectoral approach, which is associated with more extensive soft tissue handling, thereby increasing the risk of surgical site infection. One patient developed a hypertrophic scar.

The overall complication rate was around 21%, which is comparable to the rate of complications observed after fixation of proximal humerus fractures by PHILOS plating using the deltopectoral approach, as observed in the systematic review and meta-analysis by Oldrini et al. (2022) [5].

While axillary nerve palsy remains a concern, our study suggests that the overall complication rate associated with PHLP plating via the deltoid-split approach is acceptable. Non-union and deep infection were absent. The absence of screw perforation reinforces the advantage of enhanced mechanical stability offered by the locking plate system. These findings are important in establishing the deltoid-split approach as an alternative to the deltopectoral approach.

**Figure 4:**
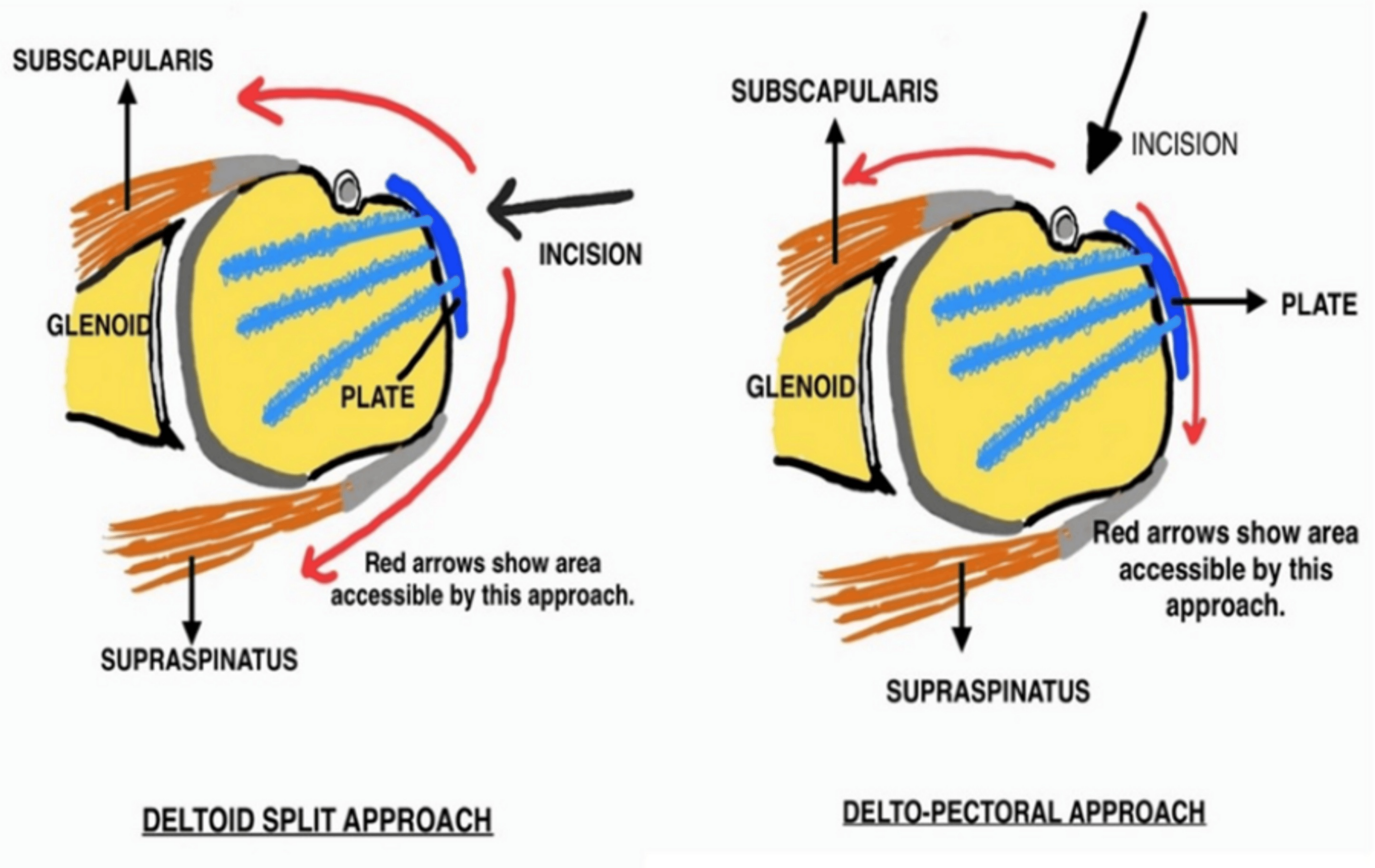
Diagrammatic representation showing the difference in access areas obtained by the two approaches (deltoid-split versus deltopectoral approach) Image credit: Dr. Rajesh Arora This image is an original author-created schematic using the Samsung Notes app (Samsung Electronics, Suwon, South Korea) on an Android tablet and was not generated using AI.

Through the deltoid-split approach, we gained direct access to the lateral aspect of the humeral head, and it was easy to reduce and fix greater tuberosity fragments. Similarly, better access to the rotator cuff for tying it with the plate was possible using this approach (Figure 4).

Subsequently, it was realised that this approach was more effective for fractures with posterior comminution, whereas, for fixing fractures with medial calcar comminution, it could be challenging, especially in cases presenting late and in those associated with dislocations (better access for fixation in cases of medial calcar comminution could be achieved by the deltopectoral method). The axillary nerve was kept protected during surgery; however, the location of the axillary nerve was invariably found to be over or around the critical calcar screw holes. This made the placement of calcar screws difficult, and extra caution had to be taken to ensure that the axillary nerve was not injured during the placement of calcar screws.

The deltoid-split approach requires less soft-tissue dissection, thereby preserving the blood supply to the humeral head and reducing the risk of AVN of the humeral head. However, one needs to be careful of the axillary nerve and be well versed in the technique of indirect reduction and the use of reduction tools such as McDonald’s retractors, K-wires, and trans-rotator cuff sutures.

The current study was a prospective case series assessing the functional outcome of proximal humerus fractures following surgery with the deltoid-split approach only. However, there was no control group in the current study. The current study also had a limited sample size, with patients operated on at a single center by a single consultant orthopedic surgeon. A comparative study with a larger sample size and multicentric design could have provided data that could have been used to assess the superiority of one approach over another in terms of functional outcomes and complications. The follow-up duration was limited to 12 months only. This is sufficient to observe and record early functional outcomes, but to evaluate long-term complications like AVN, arthritis, and implant longevity, longer follow-up is necessary. Although QuickDASH and VAS scores are useful outcome measures, they have their own fallacies and advantages. Inclusion of other scores such as the Constant-Murley score [18] and the Oxford Shoulder score [19] could have provided a more comprehensive picture of functional outcomes.

We tried to obtain the mean Constant score for our population using the study published by Chee et al. (2025) [20], wherein the formula for conversion was obtained from a population with a mean age of 69.2 ± 11.74 years, while our study population had a much lower mean age, that is, 43.3 years, a difference of over 25 years (>2 standard deviations). This placed our cohort outside the original study population, leading to extrapolation, where the predictions became less reliable. Also, the formula has limited explanatory power (adjusted R² = 0.334) and shows wide patient-to-patient variability (approximately ±31 points). Therefore, this formula, when applied to our study population, yielded irrelevant values, and we did not use this study to calculate the Constant score [20].

## Conclusions

Our study demonstrated that locking plate fixation in proximal humerus fractures via the deltoid-split approach yields good functional outcomes, as shown by improvements in QuickDASH and VAS scores between 3, 6, and 12 months. The technique is associated with a low complication rate, with axillary nerve palsy being the most common issue. It involves less soft-tissue dissection and provides good access to tuberosity fragments, making it a valuable surgical option in the management of proximal humeral fractures, especially those with greater tuberosity fractures or posterior comminution. The deltoid-split approach can be considered part of the armamentarium of a trauma surgeon for the fixation of proximal humerus fractures. 
